# Development of a New Method to Track Multiple Honey Bees with Complex Behaviors on a Flat Laboratory Arena

**DOI:** 10.1371/journal.pone.0084656

**Published:** 2014-01-20

**Authors:** Toshifumi Kimura, Mizue Ohashi, Karl Crailsheim, Thomas Schmickl, Ryuichi Okada, Gerald Radspieler, Hidetoshi Ikeno

**Affiliations:** 1 School of Human Science and Environment, University of Hyogo, Himeji, Hyogo, Japan; 2 Department of Zoology, Karl-Franzens-University Graz, Graz, Austria; 3 Kagawa School of Pharmaceutical Sciences, Tokushima Bunri University, Sanuki, Kagawa, Japan; CNRS, France

## Abstract

A computer program that tracks animal behavior, thereby revealing various features and mechanisms of social animals, is a powerful tool in ethological research. Because honeybee colonies are populated by thousands of bees, individuals co-exist in high physical densities and are difficult to track unless specifically tagged, which can affect behavior. In addition, honeybees react to light and recordings must be made under special red-light conditions, which the eyes of bees perceive as darkness. The resulting video images are scarcely distinguishable. We have developed a new algorithm, K-Track, for tracking numerous bees in a flat laboratory arena. Our program implements three main processes: (A) The object (bee's) region is detected by simple threshold processing on gray scale images, (B) Individuals are identified by size, shape and spatiotemporal positional changes, and (C) Centers of mass of identified individuals are connected through all movie frames to yield individual behavioral trajectories. The tracking performance of our software was evaluated on movies of mobile multi-artificial agents and of 16 bees walking around a circular arena. K-Track accurately traced the trajectories of both artificial agents and bees. In the latter case, K-track outperformed Ctrax, well-known software for tracking multiple animals. To investigate interaction events in detail, we manually identified five interaction categories; ‘crossing’, ‘touching’, ‘passing’, ‘overlapping’ and ‘waiting’, and examined the extent to which the models accurately identified these categories from bee's interactions. All 7 identified failures occurred near a wall at the outer edge of the arena. Finally, K-Track and Ctrax successfully tracked 77 and 60 of 84 recorded interactive events, respectively. K-Track identified multiple bees on a flat surface and tracked their speed changes and encounters with other bees, with good performance.

## Introduction

The majority of ethological studies rely on accurate observation of animal behavior. Animal behaviors have been studied by monitoring the movement of target animals in both field and laboratory environments. In such experiments, the model animals are contained in circular or rectangular arenas. Behavioral information is gathered by recording the trajectories and variation of animal movements within the arena. To date, small animals such as flies [1], mice [2]–[4], spiders [5], and cockroaches [6], [7] have been used as model animals. Social insects such as bees [8] and ants [9] are also popular for studying animal social mechanisms. In these ethological studies, necessary data on animal sociality are collected by means of video recordings and computer analysis. Recent developments in recording equipment, such as digital video cameras and webcams, provide high functionality at reasonable cost, enabling long-term movements of target animals to be captured rapidly and easily. However, although human observers can easily monitor the target animals using these recordings, extracting behavioral data from the movie images remains a laborious and time-consuming manual task. In addition, manual analyses of sequential images may yield insufficient quantitative and objective ethological data.

Recent automatic tracking programs for collecting ethological data from video images have enabled us to analyze various animal behaviors in the laboratory quickly and precisely; for example: [10]–[12]. For example, the program developed by Delcourt et al. has successfully tracked juvenile Nile tilapias (*O. niloticus*), which often swim in schools, and has identified three different crossing patterns [13]. The open-source program Ctrax, published by Branson et al. [1], was developed for tracking multiple flies walking in an arena. Ctrax has been widely used for tracking not only flies [1], [14]–[17] but also ants[18], [19], cockroaches [20]–[22] and fish [23]–[25]. These programs detect individuals by subtracting background images. The location of target animals at time *t* is estimated from a constant-velocity model, based on positional change from time zero to *t*-1. Therefore, these models are of limited applicability because target animals must move on a constant background such as an arena, and their movements are assumed continuous, streamlined and unvaried. Because of these limitations, automatic tracking remains an important challenge in the behavioral analysis of animals with diverse movements, such as ants and honeybees.

Various equipments and methods have been developed and applied for tracking animal behaviors such as flies, mice and ants. For example, the video-based systems tracked targets based on shape and/or color [26]–[28]. These characteristics are usually used to identify and track them. For more accurate identification, the barcodes were used for identification by Mersch et al. [29]

The PFID chip was also used for tracking animal behavior [30]. As same as the video-based tracking using a barcode, the animals can be identified by attached unique PFID chip. Furthermore, the fusion video-RFID tracking method was already applied by Weissbrod et al. [31]. Even the methods with physical attachments are helpful for identification; many researchers want to use the video-based tracking because of its convenience and little influence on the animals.

The waggle dance of honeybees, discovered by Karl von Frisch in 1967 [32], is one of the most famous social behaviors. By conducting this dance, the honey bee shares information of profitable food sources with her nest mates. The waggle dance has roused much interest among ethologists, rendering the honey bee a popular model animal for studying social behaviors. Other social behaviors displayed by honeybees include division of laborious tasks such as cleaning and building of combs, caring for the queen and brood, defending the hive from potential predators and controlling the moisture and temperature in the hive [33]. Adult honeybees also transfer food to other adults in their colony (trophallaxis), which serves a communicational, nutritional and transport function [34]–[37]. From the type, quality and willingness of the donation, the recipient obtains information about the food condition in the colony or within a smaller subgroup of bees. Trophallaxis is typically conducted with the bees facing each other in a line. The trophallactic strategy has been adopted in robots [38]. Although the capabilities of an individual are limited and few, the bees collectively achieve high performance. Cooperation enables the colony to survive cold winters, which individual bees could not survive, and rapidly boosts the foraging workforce in spring, when other social insects (such as bumble-bees and wasps) remain in the colony-founding phase. Monitoring and analyzing individual behaviors together with social interactions is expected to reveal the social structure and performance of a honeybee colony.

The social behaviors of bees are generally investigated on flat surfaces. Some researchers have analyzed the waggle dances on a flat vertical observation hive [39], while others have monitored the response of young bees to temperature in a flat circular horizontal arena [40]. Such flat-surface experiments are important for observing and analyzing the social behaviors of honeybees. However, automatic tracking of honeybee behavior is not readily achieved using existing computer software, because honeybees display complex and unique behaviors such as contact with other individuals and accidental movement, i.e. resting or stopping within the hive. Such behaviors require analysis by a new tracking method.

Previously, we developed a method that tracks hundreds of unmarked honey bees walking in an observation hive [41]. In this method, individuals are distinguished and tracked based on body size and shape, and the spatiotemporal overlapping of bee regions. Within a few minutes, the algorithm tracked more than 350 in a colony of about 700 bees in an observation hive. However, precise, longer-term tracking of several bees in an arena could not be undertaken without losing bees from time to time. Number retention is an important prerequisite for studying the social behavior of honeybees. Furthermore, we developed a software to track multiple bees on a flat arena [42], based on the previous algorithm [41]. The software could estimate the moving area of each individual to trace the complex behaviors of bees. Our software could detect independent central points of each bee frame by frame with nearly 95% accuracy. However, the program still has limitations to connect with these points as the movements such as the interactions between bees.

In this article, we describe an improved method for tracking unmarked multiple honeybees, based on the previous work [42]. There is the difference between previous and current programs that current program adopted two processes, predicting the linear movement or regional matching, as a method to identify individuals when they overlapped. Our method implements three major processes: (A) Regions occupied by bees are detected, (B) Individuals are identified, and (C) The behavioral trajectory of an individual is constructed, based on the known complex behaviors of bees. Overlapping individuals are identified in our program by one of two processes; predicting their linear movement or regional matching. Our new software, named “K-Track”, was validated by tracking 16 honey bees moving on a circular arena, and comparing the results with those obtained from Ctrax (0.3.9) [1], a well-known free software package for animal tracking. We demonstrate the superior tracking performance of our program, compared to “Ctrax”.

## Methods

To precisely assess the movements of an animal species, the behavioral properties of the animal must be ascertained. The main problem in simultaneously tracking multiple bees is the difficulty in identifying and separating overlapped or contacting individuals. Complex patterns arise from the combination of individual' movements, especially when three or more bees interact. We assume that, in the absence of interaction (a reasonable proposition on frame-rate time scales) a single bee moves linearly forward. However, in practice, the movement of a bee is often influenced by interactions with other bees, and the linear prediction is incorrect even on short time scales. In this case, our tracking algorithm would lose contact with the bee. As a contingency strategy for such frequent events, the neighboring regions were searched for the target bee and the target position updated by matching and detecting all local individuals.

**Figure 1:**
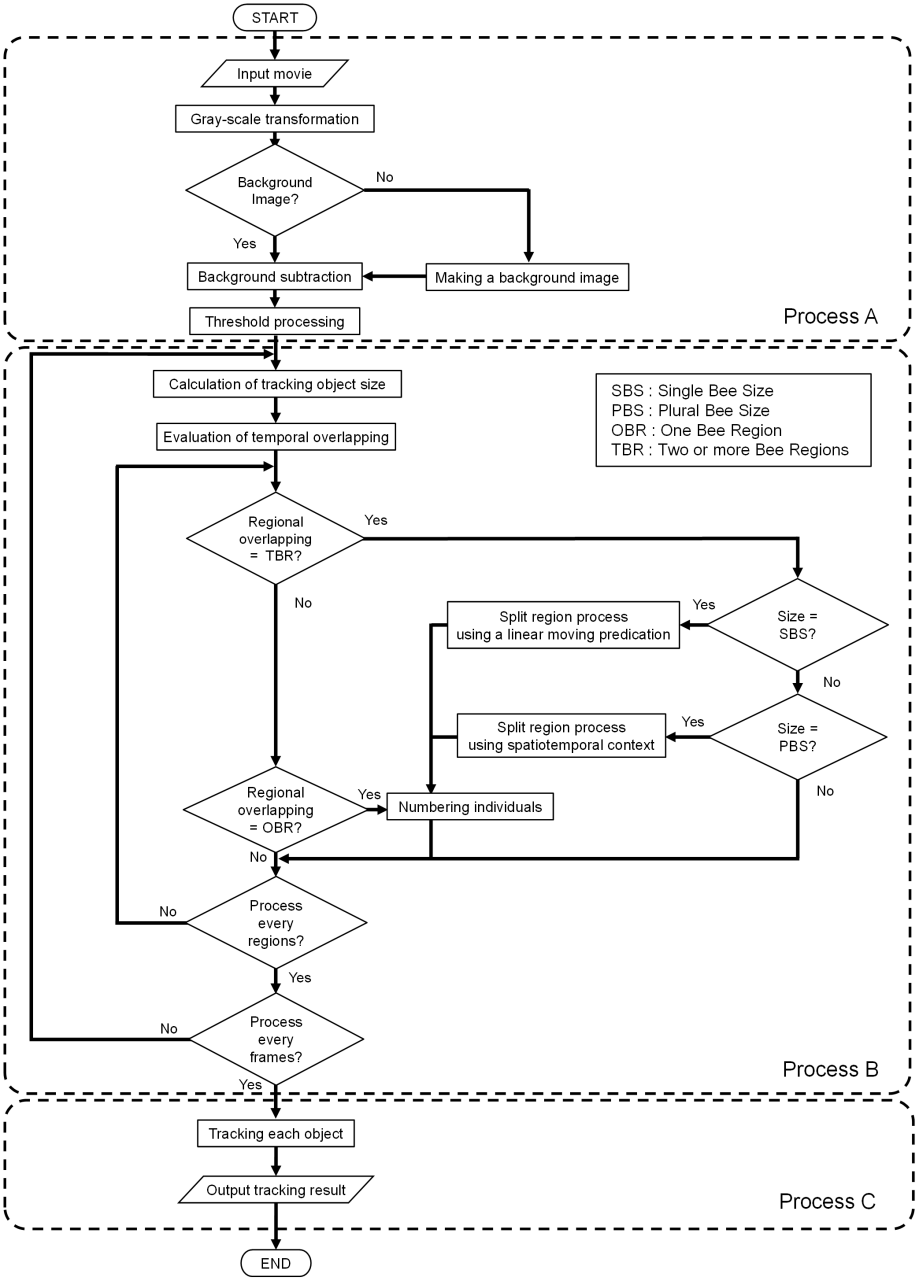
Workflow of our proposed method. The method consists of three main processes; (A) Detecting bee candidate regions using gray-scale transmission and threshold processing; (B) Identification and numbering of individuals, achieved by extracting individuals from regions containing two or more bees; and (C) Assigning *x* and *y* position coordinates to each bee, outputting the results and connecting them into trajectories.

The new method is adopted for tracking multiple bees in a movie depicting spatiotemporal changes of bee body sizes, shapes and locations. Individual bees are treated as rigid objects, distinguished and separated by “size” and “shape” when they are extracted from original images. Our algorithm can assign each tracked object a unique identification number (ID) by analyzing the temporal changes of two key aspects. The trajectory of a bee is obtained by connecting the centers of mass of individuals which are assigned specific ID numbers in sequential frames. The workflow of our proposed method proceeds as shown in Fig. 1: (A) The region occupied by an object (bee) is delineated by simple threshold processing of gray scale images, (B) Individuals are identified from spatiotemporal contextual information on size, shape and location, and (C) Behavioral trajectories are drawn by connecting the centers of mass of individuals sharing the same ID number through all movie frames.

### Extraction of a candidate bee's regions

**Figure 2:**
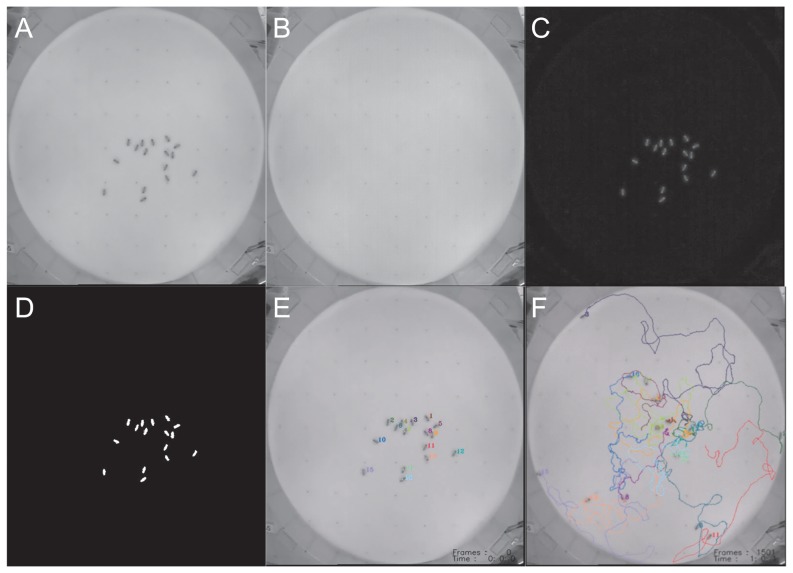
Snap shots to illustrate each step of the data analysis. (A) original image, (B) background image to make from original images, (C) the image to process background subtraction, (D) the image to process the binarization, (E) the result image of identification of bees and (F) the tracjectories of every individuals.

In process A, an image is split into two images; ‘foreground’ and ‘background’. Ideally, all tracked objects should exist in the foreground image. If the background image is obtained first, the source image (Fig. 2A) is readily divided into the two categories. If no background image is recorded before the bees enter the arena, the background must be deduced from the movie data. This is achieved as follows: the gray scale levels of both bees and background are constant under stable light conditions. In our honeybee arena, the background is brighter than the animals, so the gray scale values of bee-associated pixels are lower than those of the background pixels. The background image (Fig. 2B) is obtained by allocating the maximum gray scale value to each pixel within all movie frames. A series of foreground images (Fig. 2C) is then obtained by subtracting the generated background image from the source images. To identify the sizes and shapes of the bees, all foreground images are converted to binary images (Fig. 2D) based on a predetermined threshold.

For testing the robustness of our software, we investigated the relationship between the threshold value and the trajectory's accuracy. We used the Trajectory Completeness Factor (TCF) value [43] to express this accuracy. If the calculated trajectory of tracking software fits in the correct trajectory, the TCF value is 1. If the software fails to track a target, the TCF value is less than 1. In this study, the software binarized the images of the movie1 using the threshold value 43 and tracked the bees using this result. The TCF values between 38 and 53 of the threshold value are 1.00, showing that our software succeeded to track all bees in this range (Fig. S1). This shows that our software has strong robustness for the variation of threshold.

### Detection of a bee's regions

**Figure 3:**
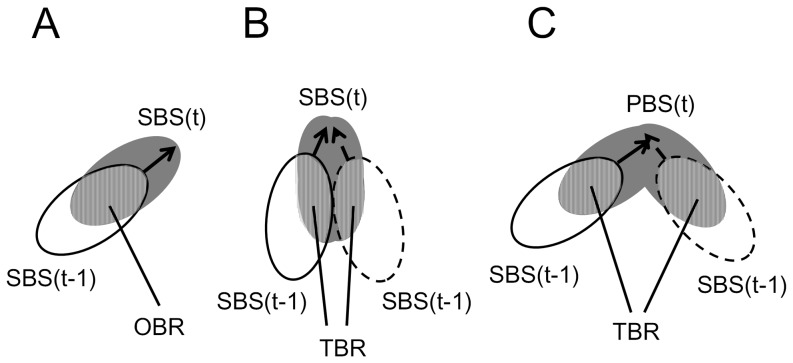
Spatial overlapping patterns are classified by size changes between current and former regions. (A) Single bee moves straight ahead. Region size is within range of single-bee size (SBS) and overlap is one-bee region (OBR). (B) Two bees appear as a merged entity in the source image. Region size is SBS and overlap is two-or-more bee region (TBR). (C) Two bees bump into each other. Region size exceeds range of single-bee size (PBS) and overlap is TBR.

Process B aims to detect and identify all bees from the previously fabricated binary images. Honey bees possess almost no distinguishing features that allow individual identification in general. They are very similar in size, shape and color. Individual differences are smaller than the discretization variability. The noise introduced by the video recording technique and poor light conditions (red light). Regardless of such difficulties, our algorithm assigns every tracked bee a unique number that holds over the entire video period. To achieve this, we assume that the size of one bee varies slightly during each run. As a pre-processing step, our method detects the bee regions from the body size of a single bee, without individually identifying it. Identifying numbers are then assigned to the bee regions to produce a prediction and identification model for bees, parameterized from each focal movie. The number of pixels in a single bee region is calculated from the initial movie frames (20 seconds; 500 frames), which also reveal the valid size range of single honey bees (RSS: Range of Single-bee Size). The RSS is an effective measure for detecting individuals in all subsequent video frames. To allow the tracking of multiple bees, our method imposes an important restriction: The regions of an individual bee at time *t* and time *t*-1 must be overlapped (Fig. 3). In other words, a bee cannot move further than its own body length in any two consecutive frames. The algorithm then categorizes all bee-associated regions by size and by spatiotemporal overlapping in the movie frame sequence.

### Identification of bee's regions

Our program defines the size and sharp of one bee. Moreover, we assume that the bee's shape remains unchanged in an overlapping situation of two bees. Therefore, the program can calculate the number of individuals and the location of each bee at current time using the current overlapping area and the location of each bee at previous time [41]. In the next step of the algorithm, bee regions are first classified into three categories based on region size; 1) Single Bee Size (SBS): the region fits within the RSS, 2) Plural Bee Size (PBS): the region is bigger than the maximum RSS and 3) No Bee Size (NBS): the region is smaller than the minimum RSS. Next, the number of regions in the previous frame overlapped on each current region is determined. 1) No overlapping Bee Region (NBR): the number of the overlapping regions is zero, 2) One overlapping Bee Region (OBR): the number is one, and 3) Two or more overlapping Bee Regions (TBR): the number is two or more. From these two characteristics (size and overlap), individual bees can be distinguished in each movie frame. In the first frame, unique ID numbers are assigned to SBS regions (Fig.2E). In subsequent frames, ID numbers are recovered as follows:

1. SBS and NBR: This region contains a single new bee. A new unique number is assigned to this region.
2. SBS and OBR (Fig. 3A): The current bee SBS(*t*) is assigned the ID number of the previous overlapping bee SBS(*t*-1).
3. SBS and TBR (Fig. 3B): The current region SBS(*t*) contains two individuals. The correct SBS(t)s are delineated by the linear motion assumption. The current SBS(*t*)s are assigned the ID numbers of the overlapped SBS(*t*-1) bees.
4. PBS and TBR (Fig. 3C): The current region PBS(*t*) is divided into two SBS(*t*)s by regional matching, using spatiotemporal contextual information between the current region and SBS(*t*-1)s. The divided SBS(t) region is assigned the ID numbers of the SBS(*t*-1) bees. [35].
5. Other cases: No processing is executed.

### Output of behavioral trajectories and locational data

In process C, the location data and the behavioral trajectory of individual bees are logged during the image processing (Fig. 2F). The bee location is the position of the center of mass of each identified bee's region. These coordinates are finally exported into a CSV-format file. From the bee location data, the velocity, acceleration and direction of the bee's movements are simultaneously determined, as well as the distance moved by individuals (estimated from temporal changes in the bees' locations). Furthermore, our method can generate visual trajectories of bee behavior by interpolating between all locations of all individuals frame by frame [41]. The tracking results are also exported as an image file, with the overlapping locus of each bee on the original frames. These quantitative values will assist the further analysis of individual and collective honeybee behaviors. The method is also applicable to many other animals that move and interact in comparable arena setups.

## Experiments and Results

### Developed environment for the software

Our tracking software, called “K-Track”, was developed in Microsoft Visual Studio 2010 (Visual C++) with the Computer Vision Library: OpenCV 2.31 on a laptop computer with an Intel Core i5 - 2.50 GHz (CPU), 8 GB (Memory), 256 GB (HDD) and Microsoft Windows 7 Professional 64bit (OS). Our software was developed as a 64 bit console application run on the 64 bit version of Windows, because a large RAM (more than 4 GB) is required to store all frame images and the individual positions over all time frames and to keep a working memory space. Larger memory space would allow researchers to realize more efficient image processing and longer-term tracking of multiple individuals.

### Set up of experimental movies

We prepared four sets of 1-minute movies (1,500 frames), named ‘movie-A’, ‘movie-1’, ‘movie-2’ and ‘movie-3’, to evaluate our software. In ‘movie-A’ the movements of software-simulated and multi-artificial agents were recorded. These agents were driven by the honeybee-inspired BEECLUST algorithm [6], [40], [44]. This movie contains relatively simple movements and was used to evaluate the basic performance of our software. The movie was played at 25 frames per second, the rate of the PAL format, and the frame size of each image was 600×600 pixels. The sizes of individual honeybees and the arena were extracted from the experimental movies, which incorporated two additional behavioral components; a bee could vary its direction by rotating its body axis, or it could suddenly stop. These behaviors were added to the original BEECLUST algorithm in order to mimic real bee's behavior.

Movies 1–3 are experimental benchmark-movies of sixteen young honeybees (*Apis mellifera* L.) walking in a circular arena (radius 30 cm). These movies were recorded by an infrared camera fixed 175 cm above the arena in the Artificial Life Lab. at the Karl-Franzens-University Graz, Austria [45]. The behaviors displayed in the movies differ widely in terms of (1) average speed of movement, (2) number of interactions between two or more individuals, and (3) long-term resting behavior. More specifically:

Movie-1: characterized by slow movement, few interactions, and periods of long-term resting.
Movie-2: characterized by moderate movement, some interactions, and no long-term resting.
Movie-3: characterized by rapid movement, many interactions and no long-term resting.

**Figure 4:**
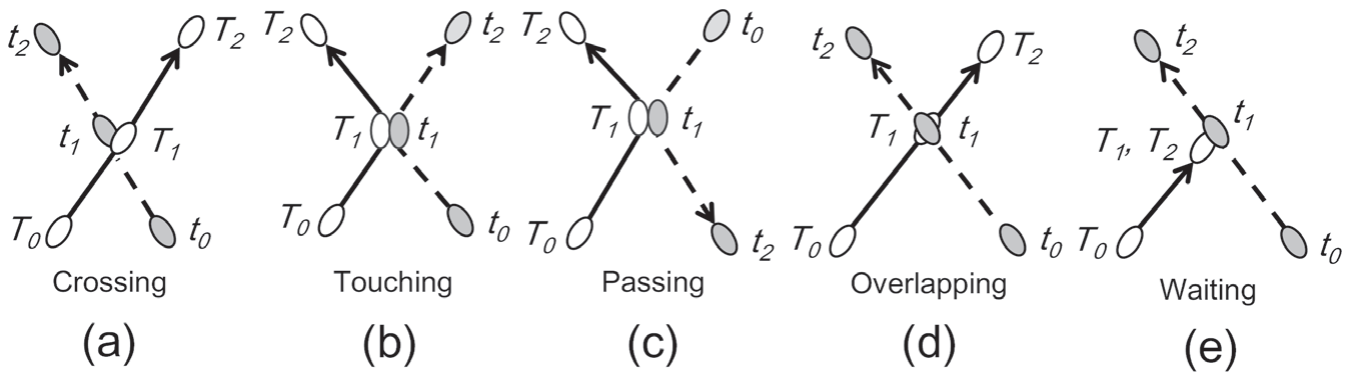
Five types of interaction patterns between two bees were extracted from the movies and classified as follows; (a) crossing: Two bees touch at time *T1* and do not change their moving directions before and after the time *T1*, (b) touching: Two bees come from same directions. They touch at time *T1* and change their moving directions before and after the time *T1*, (c) passing: Two bees come from different directions. They touch at time T1 and change their moving directions before and after the time *T1*, (d) overlapping: Two bees overlap at time *T1* and do not change their moving directions before and after the time *T1*, (e) waiting: After they touch at time *T1*, one bee keeps stopping until another bee pass by.

**Figure 5:**
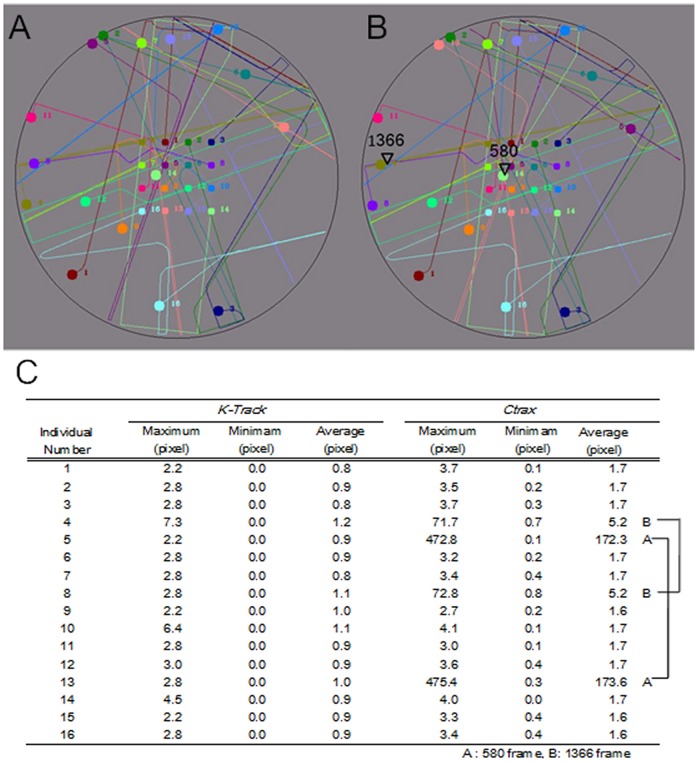
Tracking movements of bee-inspired agents in K-Track and Ctrax. (A) K-Track's trajectories. K-Track correctly identifies the positions of all agents. (B) Ctrax's trajectories. Several mistakes occur in two frames (t = 580 and t = 1366). (C) Comparison table of the average errors made by K-Track and Ctrax.

As described below, the frequency and quality of movie images (25 frames per second (PAL format) and 532×576 pixel size for the circular arena) was sufficient for tracking the behaviors of young bees in the arena. Furthermore, when evaluating our software, we paid attention to overlapping patterns, including the interactions among bees, which embody the most important social behaviors. Prior to each experiment we investigated the overlapping patterns in the arena, obtained from the experimental movies. We manually classified them into five categories; 1) crossing: Two bees touch at time T1 and do not change their moving directions before and after the time T1 (Fig. 4(a)), 2) touching: Two bees come from same directions. They touch at time T1 and change their moving directions before and after the time T1 (Fig. 4(b)), 3) passing: Two bees come from different directions. They touch at time T1 and change their moving directions before and after the time T1 (Fig. 4(c)), 4) overlapping: Two bees overlap at time T1 and do not change their moving directions before and after the time T1 (Fig. 4(d)), 5) waiting: After they touch at time T1, one bee keeps stopping until another bee pass by (Fig. 4(e)). Three of these classifications (Fig. 4(a), (b) and (d)) have been previously identified by Delcourt et al. [2]; the passing and waiting categories were deduced from our behavioral analysis of bees.

### Experiment 1 (movies of multi-artificial agents)

In the first experiment, K-Track tracked multi-artificial agents that mimic honey bee movements (movie-A). The aim of this experiment was to evaluate the basic performance of our tracking system. The algorithm performance was compared to that of the current state-of-the-art algorithm Ctrax [1]. Ctrax did not work with the default parameter values on our data. So, appropriate Ctrax parameter setting, particularly for the “Background Model”, “Background subtraction” and “Shape”, was chosen as carefully as possible. Results were obtained as a new movie containing the dynamics of the trajectories of all objects (Fig. 5). As shown in Fig. 5A, K-Track offered excellent tracking results with no misidentification, whereas Ctrax mis-tracked twice (Fig. 4B) on the same movie. We also evaluated the accuracy of position estimation by measuring maximum, minimum and average Euclidean distances between the assigned and calculated values (Fig. 4C). The average errors in object centers of mass were less than 1.2 pixels in K-Track (less than 1.0 mm in real space). In Ctrax, the average errors were below 1.7 pixels for correctly identified individuals, but the distance errors were very large because individuals were exchanged in frames 580 and 1366. Clearly, K-Track can track multiple bees on a flat surface more accurately than Ctrax (Mann-Whitney U-test, *P* = 3.443×10^−7^).

### Experiment 2 (movies recorded from honey bee experiments)

Next, the performances of the K-Track and Ctrax algorithms were tested on three honeybee movies (honeybees being the target animals of our algorithm). Interactive behaviors were the focus of this investigation. We also checked the trajectories of each individual bee. In each movie, five interaction patterns (see Fig. 4) were manually counted (Table 1). Each tracking result was evaluated by using a standard measure called the Trajectory Fragmentation Factor (TFF) and the Trajectory Completeness Factor (TCF) to measure the performance of multi-target tracking software [43], [46]. The TFF value is the necessary number of calculated trajectories to draw a correct trajectory of the target and the TCF value is the accuracy of tracking trajectory. If the calculated trajectory of tracking software fits in the correct trajectory, both TFF and TCF values are 1. If the software fails to track a target, the TFF value is more than 1 and the TCF value is less than 1. The K-Track's TFF average of all three movies is 1.29 (movie-1: 1.00, movie-2: 1.13, movie-3: 1.75). The K-Track's TCF average of the movies is 0.84 (movie1: 1.00, movie2: 0.89, movie3: 0.63). Both numbers suggest K-Track is highly accurate for tracking multiple honey bees.

**Table 1:** The numbers and errors of interaction patterns detected from experimental movies.

				K-Track		Ctrax	
			Occurrence	FalseNumber	FalseRate(%)	FalseNumber	FalseRate(%)
A	Movie1	Touching	2	0	0.0	0	0.0
		Passing	3	0	0.0	1	33.3
		Waiting	10	0	0.0	6	60.0
B	Movie2	Crossing	1	0	0.0	0	0.0
		Passing	5	0	0.0	2	40.0
		Overlapping	1	0	0.0	0	0.0
		Waiting	15	1	6.7	6	40.0
		Multiple	1	0	0.0	1	100.0
C	Movie3	Crossing	3	0	0.0	1	33.3
		Passing	18	1	5.6	0	0.0
		Overlapping	2	2	100.0	1	50.0
		Waiting	14	2	14.3	3	21.4
		Multiple	9	1	11.1	3	33.3
		Sum	84	7	8.3	24	28.6

The movements from crossing to waiting are represented in Fig. 4. The “Multiple” indicates that three or more bees interact. Failure occurs when two or more bees interact near the edge of the arena. The high false rate of the “Overlapping” state may be caused by motion rather than by the interaction pattern. Our program K-Track outperforms “Ctrax” in terms of tracking accuracy.

#### Tracking results of movie-1

**Figure 6:**
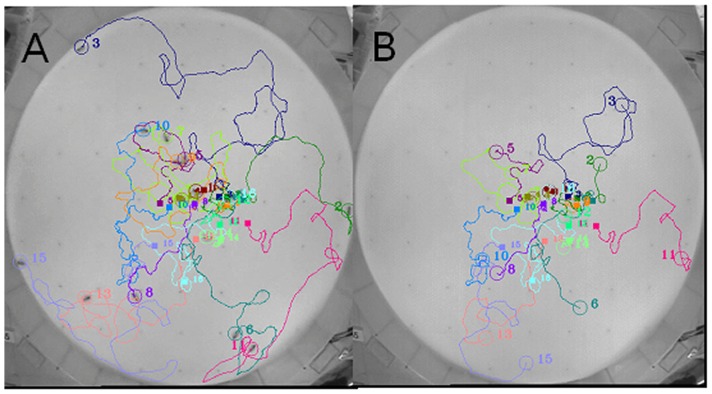
Tracking results of movie-1 by K-Track and Ctrax. The numbers shown on individuals are the identified IDs. Squares and big circles represent the start and the end points of tracking without losing the bee and re-identifying it with a new ID after the tracking. (A) Tracking results achieved by K-Track, (B) Tracking results achieved by Ctrax. The trajectories achieved by Ctrax are shorter than those by K-Track, indicating that Ctrax more frequently loses track of the bees.

First, K-Track and Ctrax were tested on relatively simple bee movements (movie-1). The bees in this movie moved slowly and interacted less than in other movies. The tracking results of the two programs are shown in Table 1A and Fig. 6. While K-Track captured all bee interaction events, Ctrax failed in 7 instances (Passing: 1, Waiting: 6). Thus, Ctrax did not always identify the behavioral states ‘waiting’ and ‘passing’, which are frequently exhibited by bees. Regarding trajectory tracking, K-Track completely tracked all movements with no duplicate ID assignments (see Fig. 6A). By contrast, Ctrax lost the movements of some individuals and could not thereafter identify them (see Fig. 6B). In this movie, some of the bees remained stationary over significant periods of time. Ctrax regarded these bees as part of the background and permanently lost their locations.

#### Tracking results of movie-2 and movie-3

**Figure 7:**
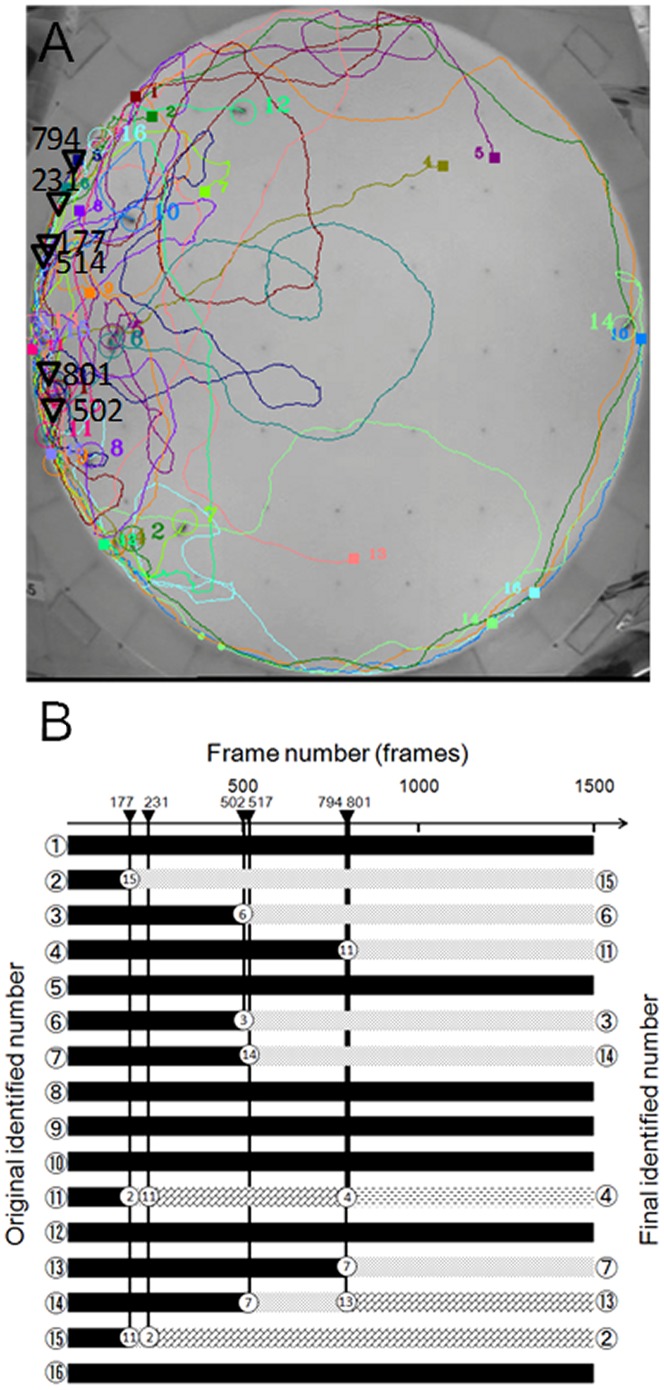
Processing of movie-1 by K-Track. (A) Trajectories produced by K-Track. Squares and circles represent start and end points of individual trajectories of each bee. Triangles show the interaction points between two or three bees. Triangles tend to aggregate around the edge of the arena, indicating that interactions frequently occur there. (B) The temporal state transition of each bee. The numbers on the inverted triangles are the frame numbers in which bee IDs were exchanged. The numbers in the right-hand circles are the exchanged IDs. Six exchanges occurred in Movie-3.

We then tested both algorithms on movie-2, which contains more complex honeybee movement patterns than movie-1. The comparison results are summarized in Table 1B. We note that K-Track made one mistake while Ctrax missed nine interaction events. As before, Ctrax tended to misinterpret ‘waiting’ and ‘passing’ states. Finally, both algorithms were tested on the third movie (movie-3) in which complex bee behavior is displayed. In this movie, K-Track and Ctrax made 6 and 8 tracking errors, respectively (see Table 1C). The positions at which K-Track fails, and the switched identification numbers and their timing, are shown in Fig. 7A and 7B. All errors occur near the edges of the arena. Our behavioral tracking algorithm assumes linear forward motion of the bees over a short period. However the circular wall forces the bees to turn and move along the curved edge of the arena. Under such conditions, K-Track cannot always correctly separate the individuals.

In summary, K-Track and Ctrax failed to separate and identify overlapped individuals in seven (8.3%) and twenty four (28.6%) interactions, respectively. Even in the middle of the arena, Ctrax failed to capture “waiting” and “passing” interactions, while K-Track could adequately process these data. Both algorithms failed around the arena edge, where linear movements are curtailed by the curved boundary. The superior performance of K-Track for tracking multiple interacting bees was confirmed.

#### Detection of interaction events

**Figure 8:**
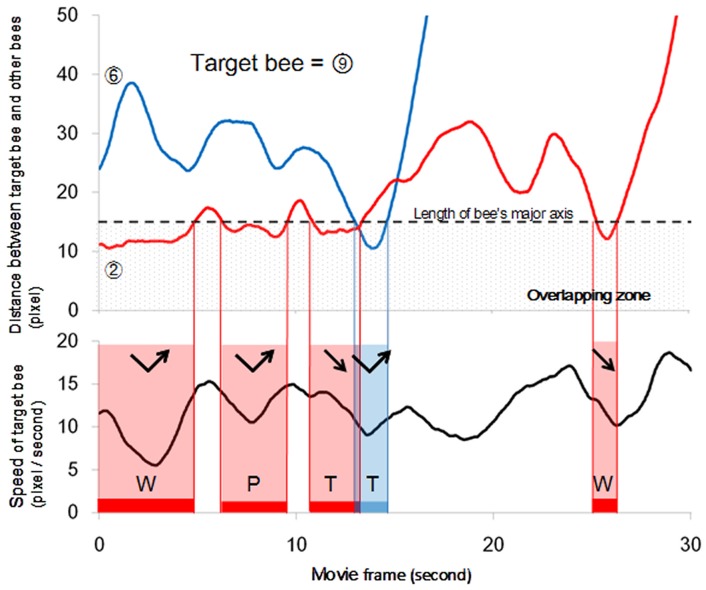
Temporal changes of distance and speed between two bees. (A) The distance between bee “ID = 9” and bees “ID = 2” and “ID = 6”, which contact bee “ID = 9”. (B) The area spans less than 20 pixels. The dotted-line shows the length of the bee's major axis. Below the dotted line, the two bees are assumed touching. In the lower part of the figure, “W”, “P” and “T” represent the identified interaction patterns “waiting”, “passing” and “touching”, respectively (see Fig. 1).

Automatic image processing and tracking has several advantages over manual image processing. For example, K-Track automatically detects the position and timing of contacts between two or more bees from the distances between individuals (Fig. 8). The interactions among multiple individuals, such as approach, contact and separation of one bee from another, are crucial for analyzing group behavior of animals. We classified such events by calculating the Euclidean distance between two bees. A bee-to-bee encounter was defined as one bee facing another at a distance of less than one body length. As an example, the Euclidean distances between target bee “9” and another colony member (“2” or “6”) were calculated at different times. The temporal changes in these distances are plotted in Fig. 8A. K-Track also calculated the velocity of bee “9” and assessed five candidates for interaction by whether the distance between individuals reduced below a specified threshold during 30 seconds (Fig. 8B). In this experiment, the threshold value was 15 pixels (the length of the major axis of the honeybee body). The walking speed of bee “9” was suddenly slowed by all five encounters, but was recovered in three cases. Thus, we confirmed that K-Track can observe detailed interactions and movements among multiple agents, and can evaluate them quantitatively.

## Discussion

K-Track demonstrates superior performance in tracking multiple honeybees compared to the current state-of-the-art algorithm, Ctrax. Among 84 crossing events observed in three experimental movies, K-Track and Ctrax successfully tracked 77 and 60 honeybee interactions, respectively. Of the two algorithms, K-Track provided higher accuracy for tracking multiple honey bees with complex crossing and contacting. We note that K-Track was specifically designed for complex honeybee interactions. Analyzing their movies of interacting juvenile Nile tilapias, Delcourt et al. [13] classified crossing events as “crossing”, “touching” or “overlapping”. In addition to these categories, we observed “passing” and “waiting” in the interaction behaviors of honey bees. Of the five main honeybee interactions, “crossing”, “touching” and “overlapping” occurred only 12 times in 84 crossing events. The majority of events (72) comprised ‘passing’ and ‘waiting’, which was not reported in Delcourt et al. [13]. To compare the results of the two algorithms in detail, we reclassified the five crossing patterns into two groups, one comprising the behavioral states “crossing”, “touching” and “overlapping”, the other holding the states “passing” and “waiting”. In the first group, both algorithms successfully tracked individuals throughout 10 out of 12 events (83.3% tracking accuracy in both algorithms). In the second group, however, K-Track achieved 93.1% tracking accuracy (67 out of 72 events), while that of Ctrax was 69.4% (50 out of 72). Such a difference in tracking accuracies indicates that conventional software is less suitable than K-track for tracking the behaviors most commonly observed in honey bee collectives. In a broader sense, this implies that K-track can more accurately track animals displaying variable crossing events.

All of the 7 tracking failures observed in K-Track occurred around the edges of the circular arena, indicating that future improvements to the algorithm should focus particularly on these regions. K-Track assumes that a bee travels ahead without changing the direction of her body axis during a crossing event. However, at the arena wall, the focal honeybee is prevented from linear movement and often bends her body near the circular edge, thereby following the curvature of the arena wall. Currently, our program does not reproduce this behavior. In future work, the movements of bees near the walls will be studied in detail to analyze the interaction patterns and the variation of moving directions in those regions. These new dynamics should be adopted into a new rule set which accounts for the special conditions at the wall. Furthermore, we plan to apply K-Track to a statistical estimation model of behavioral attributes, based on the bees' individual motion histories.

Potentially, K-Track may collect and present target interaction images for ethological studies. For example, the interaction patterns of honeybees tend to scatter throughout the movie. To extract interaction information, researchers must therefore check all frames in the movie. This manual checking is time consuming, labor consuming and error-prone, and constitutes a large problem for researchers. Our system specifies that interactions occur only when the distance between approaching bees becomes less than the bee's body size. Consequently, K-Track can easily extract only those scenes involving bee interactions, and specify the exact locations of interactions in successive frames. While animal interaction is generally regarded as a tracking problem, K-track is especially designed for such interactions and considers them in predicting the future movements of individuals. The algorithm retains individual IDs after interaction events. Because our algorithm exploits interaction data and identifies and classifies interaction events, it may greatly assist ethological honeybee research. Determining the localized interactions among clearly identified bees provides valuable information for models of pheromone exchange among bee groups. It is also useful for investigating trophallactic interactions and for analyzing the inhomogeneous distribution of social interactions in subgroups of the honeybee collectives. From the movie scenes, our software extracts the particular area in which two bees interact and identifies the bees by their ID numbers. In this way, the bees' historical behavior before the interaction event is available for automated or computer-aided analysis. Additionally, we developed an automatic program for editing specific areas in images, which can efficiently present the target area in the frames using various functions such as zooming [47]. By combining this automatic editing program with K-Track, we can extract specific areas in specific scenes containing interactive behaviors of target animals. K-Track can be automatically upgraded to collect such target images and display them effectively and emphatically. This new software should greatly benefit ethological researchers in analyzing the interactive behaviors of their target animals.

The performance of the tracking software depends on the target animals. K-Track succeeded to track the walking movements of the Argentine ants (*Linepithema humile*) [48]. It can be applied for small insects, but it still has some problems, such as animal size and frame rate of movie, in general use. We also applied K-Track for tracking grovelling behaviors of earthworms, but we failed it because of the big changes of body size during their movements.

Other tracking methods were already developed by many researchers. De Chaumont et al. used a body model of a mouse through a set of geometrical primitives linked by physical constraints to track individuals [27]. However, this software can track only two mice. Ohayon et al. used a unique back pattern of a mouse [26]. The mice have unique patterns of their backs, but the bees would not have identifiable features as mice. Freund et al. used mice with PFID transponders to detect their locations [30] and Weissbrod et al. used a method in mixing a video-based tracking and a PFID-based tracking [31]. It is easy to tag them with PFID, but is difficult to apply bees with these devices.

Branson et al. used the method that each detected fly in frame t is associated with a fly tracked in the previous frame t-1 [1]. Kabra et al. applied the Ctrax for classification of fly behaviors [49]. These methods are good performance for the flies that always move linearly, but would be not adapt for the complex behaviors of bees with long-time resting or waiting. Dankert et al. used the localized body model by fitting a Gaussian mixture model (GMM) with three Gaussians (background, other parts and body to the histogram of the values using the Expectation Maximization (EM) algorithm [28]. This method, however, track only two fries, simultaneously.

Moreover, Mersch et al. [29] developed the tracking software for multiple ants. They used the ants with ARTags to identify individuals. Similar to PFID, it is impossible to set a ARTag to each bee without a stress. Therefore, our software is quite effective to track multiple bees easily.

## Conclusions

In this paper, we proposed a novel method for tracking unmarked multiple honey bees in a flat laboratory arena, which focuses on identifying interaction events among honeybees. Based on this method, we developed a prototype software named “K-Track”. The performance of “K-Track” was compared with that of the open-source tracking software “Ctrax”. The test subjects were one movie of sixteen agents and three movies of experiments involving sixteen young bees moving in a circular arena. The proposed algorithm showed better performance in tracking multiple bees compared to Ctrax, in terms of both robustness (fewer tracking errors and losses in movies showing complex motion patterns), and richness (number of identified behavioral states) of the behavioral classifier. In future work, we plan to extend our software to handle more complex interaction patterns, such as interactions among three or more bees. Furthermore, we plan to apply our software to other social insects including ants. The current K-Track was released on INCF Software Center (http://software.incf.org/) under the BSD license.

## Supporting Information

Figure S1 **The relation between the threshold value for binarization and the TCF value.** Our software binarized the images of the movie1 using the threshold value 43 and tracked the bees. The TCF values between 38 and 53 of the threshold value are 1.00.
(TIF)

Movie S1 **Tracking result's movie of** **Figure 5** **.**
(AVI)

Movie S2 **Tracking result's movie of** **Figure 6** **.**
(AVI)
