# Supplementary figures and images for: Development of a New Method to Track Multiple Honey Bees with Complex Behaviors on a Flat Laboratory Arena
Source: PLoS One. 2014 Jan 20;9(1):e84656. doi: 10.1371/journal.pone.0084656 (PMC3896341; doi:10.1371/journal.pone.0084656)

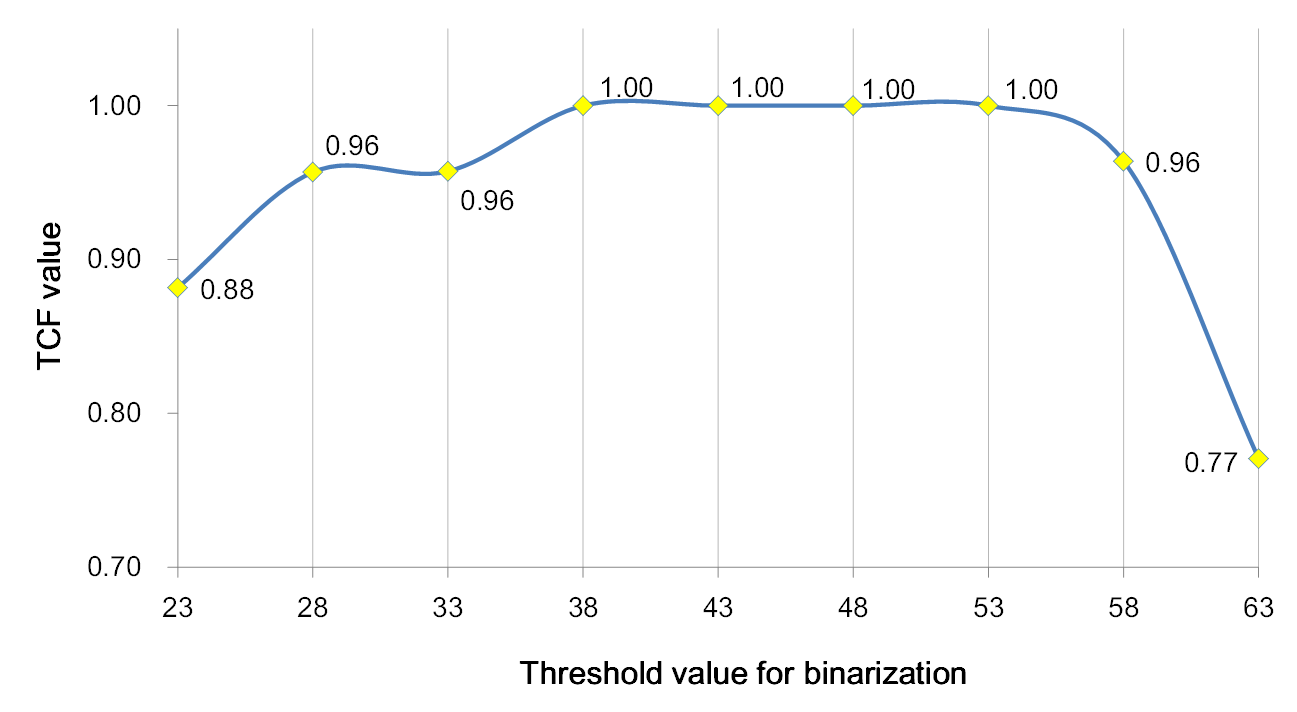

Supplement: Figure S1 — The relation between the threshold value for binarization and the TCF value. Our software binarized the images of the movie1 using the threshold value 43 and tracked the bees. The TCF values between 38 and 53 of the threshold value are 1.00. (TIF) [file pone.0084656.s001.tif]
